# Optimising the use of electronic health records to estimate the incidence of rheumatoid arthritis in primary care: what information is hidden in free text?

**DOI:** 10.1186/1471-2288-13-105

**Published:** 2013-08-21

**Authors:** Elizabeth Ford, Amanda Nicholson, Rob Koeling, A Rosemary Tate, John Carroll, Lesley Axelrod, Helen E Smith, Greta Rait, Kevin A Davies, Irene Petersen, Tim Williams, Jackie A Cassell

**Affiliations:** 1 Division of Primary Care and Public Health, Brighton and Sussex Medical School, Mayfield House, University of Brighton, Falmer, Brighton BN1 9PH, UK; 2 Department of Informatics, University of Sussex, Brighton, UK; 3 Research Department of Primary Care and Population Health, University College London, London, UK; 4 Division of Clinical Medicine, Brighton and Sussex Medical School, Brighton, UK; 5 Clinical Practice Research Datalink, the Medicines and Healthcare products Regulatory Agency, London, UK; 6 Now at: Faculty of Health and Medicine, Lancaster University, Lancaster, UK

**Keywords:** Electronic health records, Electronic medical records, Rheumatoid arthritis, Free text, Coding

## Abstract

**Background:**

Primary care databases are a major source of data for epidemiological and health services research. However, most studies are based on coded information, ignoring information stored in free text. Using the early presentation of rheumatoid arthritis (RA) as an exemplar, our objective was to estimate the extent of data hidden within free text, using a keyword search.

**Methods:**

We examined the electronic health records (EHRs) of 6,387 patients from the UK, aged 30 years and older, with a first coded diagnosis of RA between 2005 and 2008. We listed indicators for RA which were present in coded format and ran keyword searches for similar information held in free text. The frequency of indicator code groups and keywords from one year before to 14 days after RA diagnosis were compared, and temporal relationships examined.

**Results:**

One or more keyword for RA was found in the free text in 29% of patients prior to the RA diagnostic code. Keywords for inflammatory arthritis diagnoses were present for 14% of patients whereas only 11% had a diagnostic code. Codes for synovitis were found in 3% of patients, but keywords were identified in an additional 17%. In 13% of patients there was evidence of a positive rheumatoid factor test in text only, uncoded. No gender differences were found. Keywords generally occurred close in time to the coded diagnosis of rheumatoid arthritis. They were often found under codes indicating letters and communications.

**Conclusions:**

Potential cases may be missed or wrongly dated when coded data alone are used to identify patients with RA, as diagnostic suspicions are frequently confined to text. The use of EHRs to create disease registers or assess quality of care will be misleading if free text information is not taken into account. Methods to facilitate the automated processing of text need to be developed and implemented.

## Background

Electronic health records (EHRs) are a major source of data for epidemiological and health services research and service planning. Recent health policy initiatives in the both the UK and the US highlight the importance of data available within electronic health record systems [1,2]. Health policy in the UK focuses on increasing transparency of health outcomes and on quality of care, supporting greater patient choice [3]. Clinical trials may increasingly rely on electronic health records for recruitment and assessment of outcomes [4,5].

Electronic health records in the UK are most advanced in general practice (primary care) where for most practices the electronic health record is the entire health record. Electronic health records contain both structured data entered as codes (Read codes and in the past, Oxford Medical Information System (OXMIS) codes; similar to international classification of disease (ICD) codes used elsewhere in the world) and unstructured free text. Read codes are a hierarchical coding list used throughout UK general practice. Codes and text may be entered in the course of a consultation, by general practitioners (GPs) or other clinical staff such as practice nurses, or coding may be performed by administrative staff before or after the episode of care. In addition, the content of letters and other correspondence with specialists in secondary care and other health care providers can be added to the record as they are received by the practice. Sometimes an intended use of the electronic record system for research or audit is known in advance so that coding can be deliberately used to meet a set of rules or predefined codes. This will reduce the variability and standardise entry. The Quality and Outcomes Framework (QOF) rule-sets in UK primary care are an example of this [6]. QOF financially incentivises GPs to record care given for certain diseases such as diabetes and heart disease in a standardised way and is similar to the recent meaningful use initiatives in the US. However, in most primary care consultations, information is recorded by GPs for clinical and administrative purposes without consideration of its use for research or audit purposes. Hence, there may be inconsistency between GPs in choosing codes for similar cases, and thus collating information from the records is a laborious and complex process necessitating the creation of long lists of codes for each clinical entity or condition [7,8].

A comprehensive code list allows the full potential of the coded information in the records to be exploited. However, GPs may also enter information into the record as free text. The text is always associated with a code which may or may not relate to the content of the text. Using only coded information to answer research questions may miss important information which is recorded in text. Some studies have suggested coded data alone do not contain sufficient detail to evaluate clinical care or to reliably identify patient groups [9-12]. Results from our earlier study indicated that using coded data alone for case definition could potentially miss or wrongly date cases of rheumatoid arthritis [7].

However, using the free text in EHRs poses a number of challenges to researchers. The costs of anonymisation of text, to protect patient confidentiality, and the problems of using textual data in large-scale quantitative analyses mean that most research studies using EHRs ignore the information stored in the free text. Technologies to automate access to medical free text are in an early stage of development [4,6,13]. There are several possible methods for accessing the information stored in the free text, from searching manually, to automated keyword searching, to the use of more sophisticated computer algorithms such as natural language processing. Of these, keyword searching does not require researcher access to the full text and therefore avoids the need for anonymisation. It can be simply specified and quickly performed, with a keyword search giving quantitative results of how many keyword hits have been found in each patient’s record, and with which codes they were associated. However it does not allow scrutiny of text for negation, qualifiers and other context, and can only offer a rough estimation of information contained in the record. Nevertheless, as a preliminary step towards estimating the amount of information hidden in free text, it is likely to be a valuable tool. Such an approach could also be used to identify a pool of potential candidate cases that would then be reviewed manually for verification.

This study focussed on the presentation of patients with early rheumatoid arthritis (RA) in primary care. This disease was selected as an exemplar because the clinical onset is variable, the diagnosis is often uncertain in the early stages and early intervention with disease modifying anti-rheumatic drugs (DMARD) has been shown to improve prognosis [14]. There are no established code-sets for use in RA as it is not part of performance-related incentive schemes in the UK. Some EHR studies looking at other diseases, such as cancer, suggest that a diagnostic code may be recorded late in the diagnostic process, especially if the diagnosis and initiation of treatment occurs in secondary care [15-17]. In an unknown proportion of cases, a diagnostic code may not be recorded at all. We previously investigated the possibility of making a probabilistic or logical diagnosis in the absence of a diagnostic code by looking at groups of other indicators of presentation (e.g. tests, referrals, symptoms or prescriptions) [7]. We found that coded data indicating disease presentation was widespread in patient records prior to diagnosis but was unlikely to provide enough evidence to reliably identify every case. We concluded that scrutiny of information recorded in free text was needed. Some US-based studies have also found that the inclusion of automated text processing has greatly improved the precision of algorithms identifying RA cases [18,19]. The development of more sophisticated methods to identify or define cases of early rheumatoid arthritis within electronic records would facilitate service delivery and research in this disease. Here we aimed to estimate the quantity and utility of data relating to the early course of RA patients that was available within the free text section of primary care records. The objectives of this study were therefore:

1) To describe the prevalence of RA relevant keywords in free text and check for any variation by gender.

2) To estimate the quantity of information being missed when coded data alone is used.

3) To describe which codes the keywords are associated with.

4) To begin to assess the extent to which keywords can augment information in codes to contribute to probabilistic case definition.

## Methods

### Ethics statement

The study was approved by the Medicines and Healthcare products Regulatory Agency (MHRA) Independent Scientific Advisory Committee (protocol number 09_033R).

### Study population

**Figure 1:**
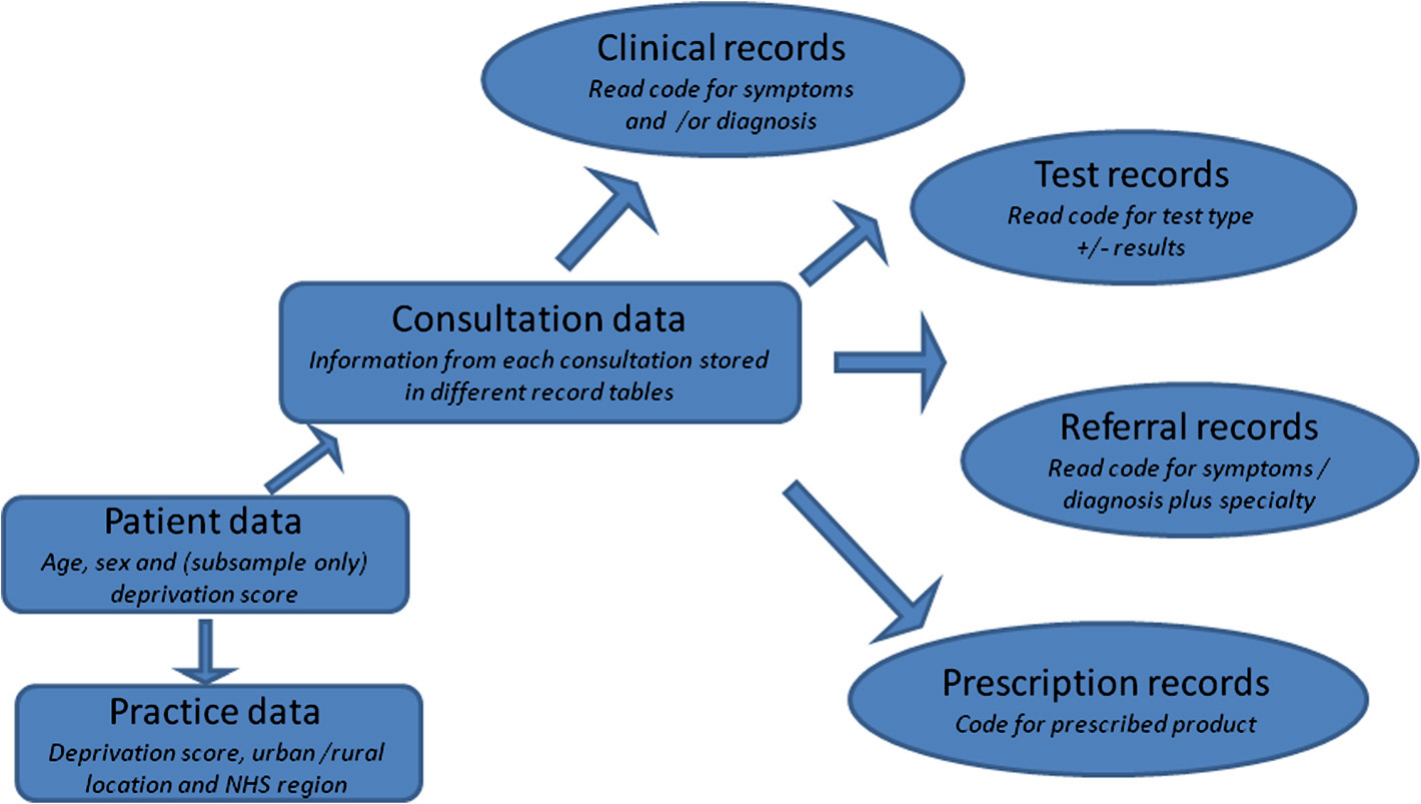
Schematic model of GPRD database.

The General Practice Research Database (GPRD) is an electronic database of anonymised longitudinal patient records from general practice (now part of the Clinical Practice Research Datalink: http://www.cprd.com). Established in 1987, it is a UK wide dataset covering 8.5% of the population, with data from over 600 practices, and is broadly representative of the UK population. There are 5.2 million currently active patients. Records are derived from the GP computer system VISION and contain complete prescribing and coded diagnostic and clinical information as well as information on tests requested, laboratory results and referrals made at or following on from each consultation. The structure of the data is shown in Figure 1, with different parts of data held in separate record tables. Each clinical event is recorded with a Read code and free text if it has been entered. Free text may be qualifiers of the codes (e.g. Code “arthritis”; free text: “Inflammatory? Rheumatoid”); notes made by the clinician in the course of the consultation (e.g. Code “patient feels well”; free text “no joint pains at the moment. Has been advised prn ibuprofen for now”); or letters of correspondence between clinicians which have been entered into the record (e.g. Code “incoming mail”; free text “Dear ~ ~~, thank you for referring this 46 year old gentleman… etc.”).

### Outcome measures

We developed two systems to work in parallel to identify events related to the presentation of rheumatoid arthritis:

1. Indicators such as tests, referrals, prescriptions or symptoms based on codes (“indicator code groups”)

2. Keywords for searching in the free text records

The categories used are summarized in Table 1.

**Table 1:** Summary of indicator markers and keyword groups

**Indicator codes – based on code**	Example codes
	(full list available in Additional file 1)
Inflammatory arthritis diagnosis	Seronegative arthritis, polyarthropathy, arthralgia of multiple joints
Rheumatoid Factor test	RhF , latex test, Rose Waller *(included regardless of result)*
Synovitis	Synovitis
DMARD prescription	List of drug names drawn up from British National Formulary
Referral to Rheumatology	Rheum. disorder monitoring, Rheum. treatment change, Rheum. management plan given, Under care of rheumatologist
Joint signs and symptoms	Joint abnormal, joint swelling, reduced joint movement, joint movement painful, joint stiffness, inflammation joint, etc.
**Keyword groups – based on text**	Example search terms
	Full list available in Additional file 2
Rheumatoid Arthritis	RA. RhA, rheum arth,
Positive rheumatoid factor test	Positive RhF , rheum fac +
Inflammatory arthritis	Polyarthitis, seronegative arthritis, inflammatory arthritis
Synovitis	Synovitis

#### Development of indicator code groups

We drew up hypothetical lists of indicator code groups based on clinical consultation and code-list dictionaries [7]. These were then modified by reviewing the codes actually used in the patients with RA before the diagnostic code was found in their records. These code-lists focussed on indicator code groups considered to be specific to RA, rather than other musculoskeletal conditions. This process, described in detail elsewhere [7], generated six indicator code groups of interest for the current study: *1) Disease modifying anti-rheumatic drug* (*DMARD) prescription, 2) referral to rheumatology*, *3) initial inflammatory arthritis diagnosis*, *4) rheumatoid factor test, 5) synovitis,* and *6) joint signs and symptoms*. Code-lists for each indicator code group as well as the list of RA diagnostic codes are available in Additional file 1.

#### Development of keyword searches

We combined three approaches to construct the keyword categories and keyword lists within each category.

1. Clinicians (rheumatology specialist & GP) drew up lists “a priori”. A rheumatologist (KAD) and two GPs (HS & GR) were asked to write down all the words specialists or GPs might use to describe a firm diagnosis of RA and a less certain diagnosis of an inflammatory type arthritis. These lists were then combined and modified to reflect the combinations of words which would be accessible in the text of the clinical and test records for the keyword search. Therefore although it was likely, as found in our previous study, that a DMARD prescription or a referral to rheumatology might be a good indicator, they were unlikely to be found within the free text in a format easily accessible or interpretable by keyword search.

2. Access to pre-anonymised text. We had access to 10,000 entries of pre-anonymised text from the GPRD from previous studies including the use of non-steroidal anti-inflammatory drugs (and not relating to the current study population). In total 1307 records which either had any one of the “a priori” terms used in codes or had the term “arthritis” in the text were reviewed. Terms in text that referred to an inflammatory arthritis diagnosis were added to the list created in stage one.

3. Use of metathesaurus. Lists were supplemented from the Unified Medical Language System Metathesaurus [20] and frequent spelling errors and abbreviations were added.

Four final categories were identified: *1) rheumatoid arthritis, 2) positive rheumatoid factor test,3) inflammatory arthritis*, and *4) synovitis*. These are summarized in Table 1 and the full keyword specification is available in Additional file 2.

### Identification of cases

From the target population of permanently registered patients in the study period of 1/1/2005 to 31/12/2008, cases were identified who had a first diagnostic code of RA within the study period, aged 30 years and over at the time of diagnosis, and who had records available from one year before the first coded diagnosis of RA to 14 days afterwards. If an event date had not been entered into the GP system, the date that the record was created was used (0.1% were imputed (10,986 events)). Events were discarded if they occurred before the start date (the latest of patient’s registration date or the date that the practice’s records were considered up-to-standard by the GPRD) or after the end date (the first of patient leaving the practice or the last date that records were received from the practice). Coded records were therefore available from one year before to 14 days after the first coded RA diagnosis.

### Keyword search

The extracted text was searched for exact string matches, and for each string of free text within the record we had a flag for whether each of the four keyword groups were present and a word count. The associated Read code was also recorded. Dummy variables were created to indicate the presence/absence of each keyword for each event in the sample. Text extraction & keyword searching were performed on the entire record back to the first of 1 year before 1st RA code, or the 1st DMARD prescription or 1st specific marker date, even if these last two extended to earlier than one year before the first RA code. Keyword searches were undertaken as simple pattern matches where the keyword sequence of characters was identified anywhere in the total free text record irrespective of word boundaries. The search was case insensitive.

### Statistical analysis

The data were prepared using Stata version 11 (Statacorp LP, Texas). For each indicator code group, any relevant code in any record table resulted in a positive hit. This was indicated in the database using categorical dummy variable for each indicator code group. The earliest code within any indicator code group or the earliest occurrence within a keyword group was used to determine the time interval prior to RA code. The Read codes associated with text strings containing keywords were examined by tabulating the frequency of codes used for different categories of keywords. The 20 most frequent codes from each category were then combined and ranked.

The prevalence of indicator code groups and keywords were calculated in men and women and compared using chi-squared tests. The time interval between the first incidence of any indicator code group or keyword and the first coded diagnosis of RA was calculated. Since the time-intervals were skewed, medians and non-parametric tests (Mann–Whitney U) were used to compare groups. Bonferroni corrections were applied for multiple comparisons.

## Results

### Study population

In total 6,387 newly diagnosed cases of RA were identified between 2005 and 2008 and were included in analyses, comprising 2,007 men and 4,380 women. Men were older (median age 62 years [inter-quartile range, IQR 51–72]) than women (60 years [IQR 49–71]; *p* < 0.001 for age difference).

### Prevalence of indicator code groups

Codes suggesting an inflammatory arthritis were present in 11% (N = 706) of patients and for synovitis in 3% (N = 179). Rheumatoid factor test, regardless of result, was recorded in code for 55% of patients (N = 3511). Codes for a DMARD prescription were found in 32% of patients (N = 2034), for a referral to rheumatology services in 38% (N = 2453) and for a joint sign or symptom in 51% of patients (N = 3234). These results are shown in Table 2.

**Table 2:** Prevalence of indicator markers and keywords in the records of rheumatoid arthritis patients in the year preceding diagnosis, and time interval before RA diagnosis

	**MEN N = 2,007**				**WOMEN N = 4,380**			
**Indicator markers**	**Prevalence of marker**		**Interval**		**Prevalence of marker**		**Interval**	
			**(days before RA code)**				**(days before RA code)**	
	**N**	**%**	**Median**	**IQR**	**N**	**%**	**Median**	**[IQR]**
Inflammatory arthritis diagnosis	237	11.8	79	[22–154]	469	10.7	71	[17–175]
Synovitis	65	3.2	66	[22–153]	114	2.6	84	[28–188]
RhF test	1,114	55.5	49	[9–137]	2,397	54.7	43	[6–127]
DMARD prescription (not including steroids)	681	33.9	45	[0–309]	1353	30.9	71	[0–326]
Referral to Rheum	811	40.4	66	[13–183]	1642	37.5	70	[11–193]
Joint sign or symptom	1032	51.4	132	[54.5-245]	2202	50.3	133.5	[52–259]
**Keywords**	**Prevalence of keyword**		**Interval**		**Prevalence of keyword**		**Interval**	
			**(days before RA code)**				**(days before RA code )**	
	**N**	**%**	**Median**	**IQR**	**N**	**%**	**Median**	**IQR**
Rheumatoid A	590	29.4	37	[0–121]	1,262	28.8	28	[0–122]
Positive RhF test	911	45.4	49	[7–136]	2,033	46.4	47	[7–151]
Inflammatory arthritis	378	18.8	70	[17–168]	790	18.0	78	[21–183]
Synovitis	379	18.9	57	[7–159]	789	18.0	55	[7–160]

### Prevalence of keywords in free text

As shown in Table 2, keywords for rheumatoid arthritis were found in 29% of patients (N = 1832). Keywords indicating a positive rheumatoid factor test were present in 45% (N = 2,944). In 18.3% of patients (N = 1168) there were words suggesting an inflammatory arthritis in their records and the same number (N = 1168; 18.3%) had keywords indicating synovitis. There were no gender differences in the prevalence of indicator code groups or keywords. Some patients had more than one keyword or more than one hit for each keyword. Of the sample of 6,387, 26.1% (N = 1668) had one keyword, 10.8% (N = 689) had 2 keywords, and 5.8% of patients had 3 or more keywords (N = 372).

### Timing of keywords in relation to codes

The indicator code groups under investigation appeared around 1 to 3 months before the RA diagnostic code was found on the record (median interval before RA code for inflammatory arthritis = 71 days (IQR = 18–164); rheumatoid factor test = 46 days (IQR = 7–147); synovitis = 78 days (IQR = 26–180)). The code category found furthest in time from the RA code was joint signs and symptoms, found a median of 133 days before the diagnostic code (IQR = 52–254). Keywords for rheumatoid arthritis were found a median of 32 days before the RA diagnostic code was added (IQR = 0–122). The intervals between keywords and RA code were similar to intervals between the indicator codes and RA code. For example the median time before RA diagnosis for a keyword suggesting inflammatory arthritis was 78.5 days (IQR = 21–184), for a positive rheumatoid factor test was 48 days (IQR = 7–147), and for synovitis was 57 days (IQR = 7–160). Intervals were similar in men and women with no statistically significant differences once corrections were made for multiple comparisons.

### Association of keywords with codes

The most frequent Read codes used in conjunction with text strings containing keywords are summarized in Table 3 (35 codes in all, the top 20 for each keyword category). *Letter from specialist* and *seen in rheumatology clinic* were the most frequent across all categories and many of the most frequent codes were not obviously related to the management of arthritis, such as *incoming mail NOS*, *patient reviewed, had a chat to patient,* and *suspected condition*. Of the top 35 codes, nine codes (26%) related to the specific indicator codes for referral to rheumatology (two codes), rheumatoid factor test (three codes), synovitis (two codes), and specific arthritis diagnoses (two codes). A further eight codes (23%) related to non-specific signs, symptoms or diagnoses (three codes for joint pain, four codes for non-specific arthritis diagnoses and one for a non-specific test). Ten of the codes (29%) were related to contact with hospital specialists, suggesting that referral, discharge or clinic letters may be a rich source of keywords.

**Table 3:** List of the most frequent Read codes used in conjunction with free text containing keywords

**Read code**	**Frequency**	**Overall rank**	**Rheumatoid arthritis rank**	**Inflammatory arthritis rank**	**Positive RhF test rank**	**Synovitis rank**
Letter from specialist	2576	1	1	1	1	1
Seen in rheumatology clinic	1979	2	2	2	2	2
Incoming mail NOS	770	3	4	3	4	3
Rheumatoid factor	592	4	3	10	3	
Rheumatoid arthritis	546	5	6	11	5	6
Patient reviewed	482	6	9	5	7	5
Pain in joint - arthralgia	467	7	11	6	8	4
Had a chat to patient	345	8		17	6	7
Rheumatoid factor screening test	313	9	5		9	
Incoming mail	253	10	13	9	13	8
History / symptoms	239	11	8		10	
Seen by rheumatologist	215	12	14	8	15	9
Communication from:	208	13	12	19	14	10
Arthritis	197	14		4	11	12
Advice to patient - subject	191	15	7		20	
MED3 - doctor’s statement	186	16	10	7	18	
Telephone encounter	169	17	18	16	12	
Arthralgia of unspecified site	90	18			17	13
Hand pain	90	19			16	14
Suspected condition	67	20	16	12		
Nursing care blood sample taken	59	21			19	
Seen in hospital out-pat.	56	22	20			17
R.A. latex test	53	23	15			
Incoming mail processing	46	24		14		15
Serum rheumatoid antigen level	44	25	17			
Letter from consultant	34	26	19			
Synovitis or tenosynovitis NOS	30	27				11
Synovitis and tenosynovitis	25	28				16
Arthropathy NOS	21	29		13		
Examination of patient	21	30				18
Wrist joint pain	18	31				19
Seen in orthopaedic clinic	18	32		15		
Arthropathy NOS	17	33				20
MED3 issued to patient	15	34		18		
Seronegative arthritis	13	35		20		

### Comparison of information in codes and keywords

The numbers of patients having either a code, or a keyword in the free text, or both, for each matching category is shown in Table 4 with results split by gender. Of the whole sample, 25.5% of patients had some information regarding inflammatory arthritis and in 14.4% of patients this was in text only (not coded). Likewise 19.7% of patients had some information on synovitis and in 16.9% this information was in text only. For a rheumatoid factor test 67.9% had some information regarding a test and in 12.9% this was in text only.

**Table 4:** Comparison of information available from codes and keywords in year preceding diagnosis in RA patients

	**MEN**				**WOMEN**			
**Topic**	**Neither**	**Code only**	**Keyword only**	**Both**	**Neither**	**Code only**	**Keyword only**	**Both**
**Inflammatory arthritis**								
**n**	1,483	146	287	91	3,278	312	633	157
**(%)**	(73.9)	(7.3)	(14.3)	(4.5)	(74.8)	(7.1)	(14.5)	(3.6)
**Synovitis**								
**n**	1,598	30	344	35	3,531	60	735	54
**(%)**	(79.9)	(1.5)	(17.1)	(1.7)	(80.6)	(1.4)	(16.8)	(1.2)
**Rh Factor test**								
**n**	646	450	247	664	1,404	943	579	1,454
**(%)**	(32.2)	(22.4)	(12.3)	(33.1)	(32.1)	(21.8)	(13.2)	(33.2)

### Combination of codes and keywords as predictors for case definition

The combinations of different keywords and codes were examined to ascertain their potential usefulness for case definition and results are displayed in Table 5. Of the six indicator codes under investigation, 88.5% of patients had one of these codes, 61% had two codes and 29% had three or more codes. When the keywords were added, 91.3% of patients had at least one code or keyword, 75.3% had two or more and 55.3% had three or more, suggesting that the keyword search increased the proportion of patients in whom a selection of codes or keywords could be used for finding cases. Table 5 also shows how many patients had an RA keyword with various combinations of other codes. A combination of RA keyword with another indicator could be considered as good evidence of an uncoded RA diagnosis. Around a quarter of patients had an RA keyword and one, (28.9%) two (27.1%), or three (23.0%) additional codes or keywords. A DMARD prescription could also be considered a strong indicator of an RA diagnosis in the absence of an RA code. Twenty-seven percent of patients had a DMARD prescription and one other code or keyword, 21.0% had a DMARD and two additional markers and 15.7% had a DMARD and three or more additional markers.

**Table 5:** Combinations of 2 or more codes and keywords and with RA keyword and DMARD prescription

**Combination**	**N (6387)**	**%**
1 or more codes	5655	88.5
2 or more codes	3894	61.0
3 or more codes	1873	29.3
1 or more codes or keyword	5834	91.3
2 or more codes or keyword	4810	75.3
3 or more codes or keyword	3533	55.3
1 or more codes and 1 or more keyword	3404	53.3
1 or more codes and 2 or more keywords	2057	32.2
2 or more codes and 1 or more keyword	2602	40.7
Combinations with RA keyword		
RA keyword and 1 or more codes	1742	27.3
RA keyword and 2 or more codes	1406	22.0
RA keyword and 3 or more codes	790	12.4
RA keyword and 1 or more other keywords	1739	27.2
RA keyword and 2 or more other keywords	838	13.1
RA keyword and 3 other keywords	236	3.7
RA keyword and 1 or more codes or keywords	1845	28.9
RA keyword and 2 or more codes or keywords	1734	27.1
RA keyword and 3 or more codes or keywords	1470	23.0
Combinations with DMARD prescription		
DMARD and 1 or more codes	1598	25.0
DMARD and 2 or more codes	1022	16.0
DMARD and 3 or more codes	474	7.4
DMARD and 1 or more keyword	1128	17.7
DMARD and 2 or more keywords	749	11.7
DMARD and 3 keywords	371	5.8
DMARD and 1 or more codes or keywords	1712	26.8
DMARD and 2 or more codes or keywords	1339	21.0
DMARD and 3 or more codes or keywords	1000	15.7

## Discussion

This study population of 6,387 RA patients provides one of the largest studies of the early presentation of RA in general practice using EHRs. Our results suggest that that the process of RA diagnosis takes time and information may be available in free text before a diagnosis is recorded as a Read code. The indicator code groups under investigation (DMARD, referral to rheumatology, joint sign or symptom, synovitis, inflammatory arthritis diagnosis and rheumatoid factor test) were found in between 3% (synovitis) and 55% (rheumatoid factor test) of patients. A previous paper discussed the findings regarding indicator code groups finding they were widespread in RA patient records prior to the diagnostic code but were unlikely to be adequate for describing the full picture of the early presentation of RA or for making up a probabilistic case definition in the absence of an RA diagnostic code [7].

Findings from the current study suggest that data stored in free text can add to our understanding of the early presentation of RA. By searching for keywords, it was found that additional information was hidden in the text. For example, keywords relating to inflammatory arthritis were present in an additional 14% of patients where coded information relating to inflammatory arthritis was absent; keywords relating to synovitis were found in an additional 17% where synovitis codes were absent, and keywords for rheumatoid factor test were found in an extra 12% of cases where codes for a test were absent. The rheumatoid factor test figures are complicated by the fact that only positive results were searched for in text. The text could have reported additional tests for which no result was recorded, or which were negative, but which were not picked up in the keyword search. This extra information occurred most often close to the time of diagnosis but was present throughout the study period. Time intervals between indicator code groups and the first RA diagnostic code were similar to intervals between the keywords and the RA code, as would be expected in the recording of the same type of information.

The Read codes associated with keywords were not readily predictable. Of the top 35 codes which had keywords in the free text associated with them, only 9 were our pre-identified RA specific indicator codes. Instead, keywords were often associated with administrative codes for referrals and letters or communications from specialists. This makes sense within the context of a disease which presents in primary care but because of diagnostic uncertainty generally results in a referral followed by confirmation of diagnosis and development of a management plan within secondary care. This association of text information with communication type codes also been found in studies of other diseases, for example ovarian cancer [21]. Much of the free text regarding these conditions is likely to be found in letters between GP and specialists which are appended to the record under more general codes.

### Strengths and limitations of our study

This study offers one of the biggest sample sizes of RA patients in the literature and allowed a detailed look at the diagnostic process in primary care which is missing from the literature. There are few publications, for example, on the proportion of musculoskeletal patients referred over time from primary to secondary care [9,22]. It is also among the first to try to quantify the amount of additional relevant information available in free text. However, a major limitation of this work is that we did not look at the text directly, due to the costs of anonymisation, and therefore were not able to allow for negation or other qualifiers surrounding keywords. It is therefore feasible that some of the occurrences of the keywords are for an absence, such as *no evidence of synovitis,* or the term relates to another person, for instance *mother had a polyarthropathy*. We may therefore be over-estimating the extent of relevant information held in text. One study for example [23] found that specificity of case finding dropped from 98.2% to 38.3% when negation terms were not included in the text search. It should be noted, however, that the presence of the keyword indicates that an inflammatory arthritis is being considered or discussed with the patient, and the clustering around the time of diagnosis suggests that many of these terms will apply to the patients. Even if only half of the keywords occurring in patients without any indicator markers were related to the actual presence of, for example, synovitis in the patient, this would still increase the prevalence of synovitis by more than 8%. Despite the lack of qualifiers and negation, automated keyword searching could also be a useful tool for selecting a smaller set of cases whose records could then be manually scrutinised for specific terms.

The selection of codes for the indicators and the keywords for the searches is critical to the validity of this work. The development of the indicator markers was a rigorous process that has been described in full elsewhere [7]. Similarly we tried to triangulate the information we used when preparing the keyword lists in order to allow for as many alternative expressions and misspellings as possible. One possible explanation for the extra information in text is that we selected the wrong codes for the disease indicators, thereby missing important coded information. However, from the association between keywords and communication/letter codes as well as sick note codes (e.g. *MED3 – doctor’s statement*) it seems that information is often put in text alongside a more generic code. The process of entering communication received from hospitals is not managed in a standard way by GP practices. Sometimes letters are scanned and added to the records as a *pdf* file and therefore are not searchable in the database. In other cases the entire letter is entered into the free text section and can be searched. Another issue is that the transmission of free text from the practice to the GPRD can be suppressed by the GP using a double backslash at the start of the entry. This is unlikely to affect letters, but results in an unknown amount of free text relating to clinical consultations being withheld, again affecting estimates of the amount information available. There are therefore likely to be practice-level differences in the availability of the free text which will again lead to an under-estimation of the keywords but also has implications for technologies to increase access to textual data. It would also be worth extending the keyword list to include other indicators such as DMARDs and referrals and further work will include these in searches of free text.

For free text information entered by GPs in the course of their consultation, there is likely to be a wide range of ways to express similar concepts and it is known that many entries have spelling errors or use abbreviations. We only picked up the most frequent misspellings and abbreviations in the keyword specifications. This would lead to an under-estimate of the occurrence of keywords in the record. A full exploration of the free text by hand is planned and will help us to understand more about how information is entered by GPs in the course of their consultations, including understanding more fully the range of abbreviations used and the different ways that signs and symptoms may be described. Qualifiers and negation will be taken into account during this process, resulting in a highly accurate estimation of the information held in free text about RA presentation and symptoms.

A further limitation of this study is that we have not yet investigated how often these keywords occur in control data, that is, in patients with no RA diagnostic code. There is a theoretical possibility that the distribution of these keywords would be the same in control cases as it is for RA cases. Future work will address this possibility by comparing rates of indicators and keywords in control data to ascertain their predictive value for finding cases of RA.

### How results fit with other literature

Other authors have also highlighted the potential deficits from coded data in epidemiological studies [9]. Using live clinical data such as the GPRD for epidemiological studies requires mass application of case identification criteria, rather than examining each case individually. This can lead to high, or unknown, rates of misclassification of cases [10], which bias the outcome of studies, especially those examining rates of certain tests or treatment. Studies which define cases using only diagnostic codes may miss cases where the diagnostic information is held in free text or coded several weeks after the diagnosis has been received. A further issue is the unknown quality of consultation recording and coding which is poorly established in the literature. It appears this may vary both between practitioners and practices but also between diseases [11]. GPs may regularly use the codes most readily available in the system even if they are inappropriate, and express the clinical details in free text descriptions [11]. Free text has been used for case finding and to assess quality of care in complex conditions such as diabetes and cancer [24,25]. Several authors have shown that including data from free text increases case ascertainment for both acute conditions such as respiratory infections and chronic diseases such as angina [26-28] as well as RA [29] and can enhance estimates of symptoms in cancer presentation by 40% [30].

Ethnographic studies have the potential to help understand how social practices shape the records we used for research [31]. We need field studies on the use electronic record systems, in order to understand why coding and free text are used as they are. Records are not created by a single person but rather by collaborative work practices that are carried out for complex reasons [32]. There is additionally a tension between the use of records by health-care providers who value flexibility and expressivity, and those of researchers who value structure and categorisation [33]. Early findings from the human-computer interaction work-strand of our project show that doctors often choose not to use specific diagnostic codes early in the disease process. Sometimes there is clinical uncertainty, but sometimes coding structures do not facilitate the recording of precise clinical findings and doctors need “exit strategies” to be able to report unexpected clinical exceptions [34]. Doctors’ concerns are more centred on creating records that are useful to them and their team at the point of care, rather than on creating records that will be accessible for secondary uses. There are a number of influences that affect the degree of coding used and choice of codes and these operate at policy, local, system and individual levels.

### Implications of our findings and further work

We deliberately chose a complex non-incentivised condition which posed a considerable challenge to recording in code, so our findings may not be generalisable to other more clear-cut or incentivised conditions. A systematic review of quality of coding suggested that completeness of coding may be related to distinctiveness of diagnosis [11]. Our results lead to speculation that cases may be missed if coded data alone were used to identify patients with possible rheumatoid arthritis, before a definitive diagnosis is recorded. For epidemiological studies, an estimate of false negatives (that is, patients with the disease but not identified by the case finding algorithm) is useful to give an indication of bias within the study [10]. Including free text in case finding algorithms may increase the potential for identifying patients without diagnostic codes in these studies, thereby reducing bias. If so, it becomes imperative that systematic ways of automatically extracting and assessing information in free text are developed.

We found no evidence of differences between men and women in the balance of coded and textual data or in the timing of recording. Hence, although data based on codes may be incomplete, in this initial investigation there was no evidence of biased recording by gender or timing. This needs to be explored for other patient characteristics. The possibility of systematic differences in the way information across social groups or different co-morbidities is recorded remains and would have important implications for secondary use of such clinical databases.

The greatest hurdle to the more widespread use of text is the technological challenge to automate or semi-automate processing. We have laid the basis for methods that will allow us to further investigate extracting information concealed in free text. It is of interest that much of the keyword information was found in letters from specialists and other referral communication type text. Letters are much easier to process using computer algorithms than GPs’ clinical notes due to fewer idiosyncrasies and abbreviations in the language used, although consultation notes will still need to be scrutinised for extracting information such as presenting symptoms [25]. In future work we will add negation detection algorithms and model the context in which the keyword occurs, as well as expanding the indicators which are searched for in free text. We have obtained promising initial results in pilot experiments into deriving abbreviations and synonyms of indicators, using unsupervised machine learning techniques [35]. Other groups have had success with various text-processing algorithms in identifying RA cases and have even found these algorithms are portable between settings [18,19]. We will also investigate methods to automate the process of augmenting the initial keyword list using sample data and resources like UMLS. Once full information has been extracted from the free text, we will apply statistical methods such as cluster analysis to combinations of coded and textual information to estimate which are the best to use for probabilistic case definition for RA. These search algorithms can then be tested on “control” data where no diagnostic code for RA exists, to verify their ability to find cases using contextual information. These methodologies may extend to other complex, non-incentivised diseases and may be useful for case definition in general for studies using EHRs.

## Conclusions

The results of the current study suggest that additional information is available in free text and that this would make a useful supplement to coded information in probabilistic case definition. The use of EHR data in creating disease registers or to assess quality of care may be subject to bias if free text information is not taken into account in case-finding algorithms. Scrutiny of the full free text currently comes at a high cost in terms of anonymisation and researcher time. Automating the extraction of information from free text may help to provide additional information to maximize the utility of EHRs for research purposes.

## Competing interests

From March to August 2011, AN worked for the Cardiff Research Consortium, which provided research and consultancy services to the NHS, academia and the pharmaceutical industry. Cardiff Research Consortium had no connection with or specific knowledge of AN’s work with BSMS. The authors declare that they have no other competing interests.

## Authors’ contributions

EF and AN developed methods, carried out analysis and interpretation of results and drafted the manuscript. RK and JC developed keyword extraction methods and interpreted the results. ART was responsible for the overall project design and advised on the methods and statistical analysis. IP also advised on statistical and analytical methods. LA advised on real world GP coding issues and interpreted results. HES, GR and KAD advised on clinical aspects and interpreted results. TW advised on data identification and extraction. JAC was responsible for the overall project design and supervision, advised on methods and carried out analysis. All authors commented on successive drafts, and read and approved the final manuscript.

## Pre-publication history

The pre-publication history for this paper can be accessed here:

http://www.biomedcentral.com/1471-2288/13/105/prepub

## Supplementary Material

Additional file 1 Final Code Lists. Click here for file

Additional file 2 List of keywords for diagnosis of rheumatoid arthritis. Click here for file
