# Supplementary material for: Optimising the use of electronic health records to estimate the incidence of rheumatoid arthritis in primary care: what information is hidden in free text?
Source: BMC Med Res Methodol. 2013 Aug 21;13:105. doi: 10.1186/1471-2288-13-105 (PMC3765394; doi:10.1186/1471-2288-13-105)
Supplement: Additional file 2 — List of keywords for diagnosis of rheumatoid arthritis. [file 1471-2288-13-105-S2.docx]

**List of keywords for diagnosis of rheumatoid arthritis**

Where words / phrases can be at the **start** of the word and not be a distinct word it is indicated with a wild card * - so that arth* could include arthritis, arth, arthropathy.

**Firm diagnosis**

1. Rheumatoid arthritis category

rheumatoid arth*

rheumatd arth*

rheumat arth*

rheum arth*

rheu arth*

rhuematoid arth*

rhuematd arth*

rhuemat arth*

rhuem arth*

rhue arth*

Rh arth*

Rh. arth*

rheumatoid dis*

rheumatd dis*

rheumat dis*

rheum dis*

rheu dis*

rhuematoid dis*

rhuematd dis*

rhuemat dis*

rhuem dis*

rhue dis*

Rh dis*

Rh. dis*

# Case-sensitive starts

R A

Rh A

RH A

RA

RhA

RHA

R.A.

Rh.A.

RH.A.

R. A.

Rh. A.

RH. A.

# end of case sensitive

2. Seropositive arthritis category

seropositive arth*

seropos arth*

sero+ve arth*

sero+ arth*

sero positive arth*

sero pos arth*

sero +ve arth*

sero + arth*

sero-positive arth*

sero-pos arth*

arthritis seropos*

arthropathy seropos*

arth seropos*

arthritis sero pos*

arthropathy sero pos*

arth sero pos*

arthritis sero-pos*

arthropathy sero-pos*

arth sero-pos*

arthritis sero+ve*

arthropathy sero+ve*

arth sero+ve*

arthritis sero +ve*

arthropathy sero +ve*

arth sero +ve*

3. Positive rheumatoid factor category

rheumatoid pos*

rheum factor pos*

rheumatoid factor pos*

rhuematoid pos*

rhuem factor pos*

rhuematoid factor pos*

rheumatoid +*

rheumatoid factor +*

rheum factor +*

rhuematoid +*

rhuematoid factor +*

rhuem factor +*

RhF pos*

RF pos*

Rh F pos*

R F pos*

Rh.F. pos*

R.F. pos

Rh. F. pos*

R. F. pos

RhF +*

RF +*

Rh F +*

R F +*

Rh.F. +*

R.F. +*

Rh. F. +*

R. F. +*

latex pos*

latex +*

positive rheumatoid

positive rheumatoid fact*

positive rheum fact*

positive rhuematoid

positive rhuematoid fact*

positive rhuem fact*

positive RF

positive R F

positive R.F.

positive R. F.

positive RhF

positive Rh F

positive Rh.F.

positive Rh. F.

pos rheumatoid

pos rheumatoid fact*

pos rheum fact*

pos rhuematoid

pos rhuematoid fact*

pos rhuem fact*

pos RF

pos R F

pos R.F.

pos R. F.

pos RhF

pos Rh F

pos Rh.F.

pos Rh. F.

+ve rheumatoid

+ve rhuematoid fact*

+ve rheum fact*

+ve rhuematoid

+ve rhuematoid fact*

+ve rhuem fact*

+ve RF

+ve R F

+ve R.F.

+ve R. F.

+ve RhF

+ve Rh F

+ve Rh.F.

+ve Rh. F.

+ rheumatoid

+ rheumatoid fact*

+ rheum fact*

+ rhuematoid

+ rhuematoid fact*

+ rhuem fact*

+ RF

+ R F

+ R.F.

+ R. F.

+ RhF

+ Rh F

+ Rh.F.

+ Rh. F.

**Less certain diagnosis**

4. Inflammatory arthritis category

inflammatory arth*

inflamm arth*

inflamm. arth*

inflam arth*

inflam. arth*

infl arth*

infl. arth*

early inflammatory arth*

early inflamm arth*

early inflamm. arth*

early inflam arth*

early inflam. arth*

early infl arth*

early infl. arth*

inflammatory polyarth*

inflamm polyarth*

inflamm. polyarth*

inflam polyarth*

inflam. polyarth*

infl polyarth*

infl. polyarth*

inflammatory poly arth*

inflamm poly arth*

inflamm. poly arth*

inflam poly arth*

inflam. poly arth*

infl poly arth*

infl. poly arth*

# Case-sensitive starts

IA

I A

I.A.

I. A.

EIA

E I A

E.I.A.

E. I. A.

# end of case sensitive

5. Sero-negative or reactive arthritis category

seronegative arth*

seroneg arth*

sero-ve arth*

sero negative arth*

sero neg arth*

sero -ve arth*

sero-negative arth*

sero-neg arth*

arthritis seroneg*

arthropathy seroneg*

arth seroneg*

arthritis sero neg*

arthropathy sero neg*

arth sero neg*

arthritis sero-neg*

arthropathy sero-neg*

arth sero-neg*

arthritis sero-ve*

arthropathy sero-ve*

arth sero-ve*

arthritis sero -ve*

arthropathy sero -ve*

arth sero -ve*

reactive arth*

react arth*

arthritis react*

arthropathy react*

arth react*

6. Polyarthritis category

polyarth*

poly arth*

systemic arth*

7. Synovitis category

synovitis
